# Geospatial Disparities in Access to Outpatient Physical and Occupational Therapy Services in Texas: Implications for Health Equity and Rehabilitation Workforce Policy

**DOI:** 10.3390/ijerph23040517

**Published:** 2026-04-17

**Authors:** Madeline Ratoza, Rupal M. Patel, Wayne Brewer, Katy Mitchell, Julia Chevan

**Affiliations:** 1 College of Rehabilitation Sciences, University of St. Augustine for Health Sciences, Austin, TX 78739, USA; 2 School of Physical Therapy, Texas Woman’s University, Houston, TX 77030, USA; rpatel@twu.edu (R.M.P.); wbrewer@twu.edu (W.B.); 3 School of Physical Therapy, Texas Woman’s University, Denton, TX 76209, USA; kmitchell@twu.edu; 4 School of Health Sciences, Springfield College, Springfield, MA 01109, USA; jchevan@springfieldcollege.edu

**Keywords:** rehabilitation services, physical therapy, occupational therapy, health services accessibility, geospatial analysis, rural health disparities, workforce distribution, health policy

## Abstract

**Highlights:**

**Public health relevance—How does this work relate to a public health issue?**

- Geographic accessibility to health services is a core determinant of population health, influencing whether individuals can obtain timely rehabilitation needed to maintain function, participation, and quality of life.
- This study addresses persistent urban–rural and within-city inequities in access to outpatient physical and occupational therapy services, highlighting structural and spatial barriers that contribute to health disparities across communities.

**Public health significance—Why is this work of significance to public health?**

- By applying statewide geospatial methods to identify regions of high and low rehabilitation accessibility, the study provides actionable evidence on how workforce distribution and transportation infrastructure shape inequitable access to essential health services.
- Rehabilitation services are critical for managing disability and chronic conditions; therefore, identifying regions with prolonged travel times and low provider availability helps to clarify the mechanisms through which structural inequities may lead to delayed care, poorer recovery, and reduced community participation.

**Public health implications—What are the key implications or messages for practitioners, policy makers and/or researchers in public health?**

- Policymakers and health system planners can use tract-level accessibility findings to guide targeted workforce incentives, clinic placement, tele-rehabilitation expansion, and transportation investments aimed at improving equitable service delivery in underserved rural and peripheral urban areas.
- For researchers and public health practitioners, the study demonstrates the value of integrating GIS-based accessibility measures into health equity surveillance and workforce planning to support data-driven interventions that reduce geographic barriers to care.

**Abstract:**

Equitable access to rehabilitation services is essential for individuals living with a disability, yet geographic disparities in outpatient rehabilitation care remain understudied. This study examined spatial accessibility to outpatient physical and occupational therapy services across Texas to identify regional inequities and inform workforce and policy planning. A descriptive cross-sectional geospatial analysis was conducted using outpatient clinic location data from the Texas Health and Human Services database (2022) and population data from the 2020 U.S. Census. Clinic addresses were verified and geocoded. Accessibility was measured using an origin–destination cost matrix to estimate the travel time to the nearest clinic, and the two-step floating catchment area (2SFCA) method to calculate an accessibility index. Spatial clustering of access was assessed using the Getis-Ord Gi* statistic to identify hot and cold spots. The analysis included 2255 outpatient rehabilitation clinics across 6896 census tracts. Travel times varied substantially, with rural areas experiencing the longest travel burdens. The 2SFCA analysis revealed pronounced disparities, with low-accessibility clusters concentrated in rural and border regions and high-accessibility clusters in urban metropolitan areas. These findings demonstrate persistent geographic disparities in outpatient rehabilitation access across Texas, suggesting the need for targeted workforce placement, transportation investment, and policy interventions to improve equitable access.

## 1. Introduction

Access is defined as the timely use of personal health services to achieve the best health outcomes [1] and is shaped by key determinants such as insurance coverage, services, timeliness, and workforce supply [2,3]. Penchansky and Thomas’ Theory of Access originally described five dimensions of access (availability, accessibility, accommodation, affordability, and acceptability) as the key determinants of whether patients can realistically receive care [4]. Saurman later expanded this framework by adding a sixth dimension, awareness, to better capture patients’ knowledge of available services and how to access them [5]. An accessible service, as defined by the Penchansky and Thomas model, includes services that are “within reasonable proximity to the consumer in terms of time and distance” [4].

Rehabilitation services, including physical and occupational therapy, play a crucial role in managing disability and improving health. Access issues to rehabilitation therapies have received less attention compared to other health services. Even in the US states with direct access legislation, which allows patients to seek rehabilitation care without a physician referral [6,7], geographic barriers can hinder realized access. Realized access is defined as an individual’s actual use of healthcare [8,9]. Insurance expansions under the Affordable Care Act reduced the number of uninsured individuals nationally [10]; however, disparities in healthcare access persist, especially across rural regions that have seen a significant reduction in community and rural hospitals [11] and in states like Texas that did not expand Medicaid [12]. Geographic and structural barriers, defined as obstacles created for certain groups through policies, procedures, and institutional structures, may differentially shape access to rehabilitation services [13], further exacerbating disparities. Additionally, the passing of H.R.1—One Big Beautiful Bill Act [14] in 2025 has the potential to reverse access gains made with the Affordable Care Act. H.R.1, for example, will modify the eligibility for Medicaid to include a “community engagement requirement” mandating covered individuals to spend at least 80 h monthly at work, volunteering, or in school. While these changes will not take place until 2027, they have the potential to limit the number of individuals that are eligible for coverage, reducing access [15].

Previous studies highlight these access-related challenges: neighborhoods with higher social vulnerability were found to have fewer outpatient physical therapy clinics in Denver, CO [16]. Similarly, a pilot study using census data from the Greater Brisbane, Australia region, found rehabilitation services clustered in areas with a lower prevalence of disability [17]. Lakhani et al. used GIS mapping to understand the distribution of individuals with disability and travel times to disability services including physical, occupational, and speech therapies [18]. They found that rural regions with fewer people with a disability experienced longer travel times to care clinics [18].

Texas provides a critical context for this analysis. Texas has the largest rural population in the U.S., spanning 90% of its land area [19,20]. Rural populations experience poor access to metropolitan regions and unsafe road conditions [21,22]. Even within metropolitan areas, there exist considerable “transit deserts” particularly in the metropolitan regions of Houston and San Antonio where demand for public transportation exceeds supply due to population size [23]. On average, Texans commute 27 min to work, with only 17 percent using public transportation [24,25]. Seventeen percent of residents live in rural regions, and nearly 30% of adults report having at least one disability [19,20,26], emphasizing the importance of rehabilitation services. Texas’ demographic diversity, transportation challenges, and uneven healthcare infrastructure make it an ideal setting to explore spatial accessibility. Additionally, a recently published study investigated the availability of physical therapy and occupational therapy providers in Texas and found substantial geographic variability in rehabilitation provider supply across census tracts in Texas, with lower availability in areas with populations with greater needs, including those with higher rates of disability [27].

The purpose of this study was to (1) examine spatial accessibility to outpatient physical and occupational therapy services across Texas, a large and demographically diverse state, and (2) to identify regional disparities to inform workforce and policy planning. Geospatial analysis and geographic information systems (GIS) were employed to provide insights into rehabilitation accessibility. This study extends prior rehabilitation workforce research by moving beyond provider availability alone to characterize how travel burden, service concentration, and neighborhood-level clustering shape inequities in realized geographic access across both rural communities and major metropolitan regions. By examining these patterns at the census tract level across an entire state, this work provides a descriptive, applied assessment of how structural and spatial barriers may differentially affect access to rehabilitation services.

## 2. Materials and Methods

### 2.1. Study Design and Setting

This study used a descriptive cross-sectional geospatial analysis method, integrating data on the locations of outpatient PT and OT clinics with population data from the 2020 United States Census. The merged datasets were analyzed to explore population factors associated with spatial accessibility.

### 2.2. Data Sources and Study Population

Data on clinic locations were obtained from the Texas Health and Human Services (THHS) licensure database [28] in September 2023, representing active practice sites for licensed physical and occupational therapists as of September 2022. Population and demographic characteristics were extracted from the 2020 American Community Survey (ACS) [29] at the census tract level.

The mailing list of licensed physical and occupational therapists was obtained from the Executive Council of Physical Therapy and Occupational Therapy Examiners (ECPTOTE) through an open records request submitted on 30 November 2022 [30]. This dataset provided residential addresses for all actively licensed PTs and OTs as of November 2022. Community-level demographic characteristics, including population size, race and ethnicity, disability prevalence, and poverty indicators, were drawn from the 2020 American Community Survey (ACS) [31]. Data cleaning, GIS workflows, and calculation procedures for provider-to-population ratios followed standardized methods previously developed by the research team and described in detail in a recently published feasibility study [32]. Unlike our prior provider-level workforce analysis, the present study used verified unique outpatient clinic locations as the analytic unit, enabling facility-based accessibility modeling [27,32].

### 2.3. Data Preparation

Clinic address data extracted from the Texas Health and Human Services (THHS) licensure database in September 2023 were standardized, verified through Google Maps and clinic websites, and geocoded in ArcGIS Pro (v.3.3, ESRI) [33] using the World Geocoding Service [34]. Duplicate addresses representing the same practice site were identified and removed. Because the licensure dataset captures provider-level practice addresses rather than unique facility identifiers, multiple licensed therapists practicing at the same outpatient site generated repeated records. Manual verification using clinic websites and Google Maps was used to reconcile naming variations and confirm unique outpatient facilities. Only outpatient facilities that provided direct patient care were included; administrative offices, inpatient facilities, and home health agencies were excluded. The total number of clinics identified in this way was 2255. Census tract boundaries and demographic estimates were obtained from the 2020 American Community Survey (ACS) at the tract level (n = 6896 tracts). A detailed data cleaning workflow, duplicate counts, and verification steps are provided in the Supplementary Materials.

### 2.4. Geospatial Analysis

An origin–destination (OD) cost matrix analysis in ArcGIS Pro (v.3.3, ESRI) was used to determine the travel time to the nearest outpatient rehabilitation clinic for each Texas census tract centroid. Centroids (n = 6896) were generated from census tract polygons using the “Feature to Point” tool, providing a consistent origin for analysis. The destination dataset consisted of 2255 verified outpatient physical and occupational therapy practice sites imported and geocoded in ArcGIS Pro.

A high-quality routable network dataset was essential for accurate travel time modeling. Publicly available Texas road network files lacked elevation and routing attributes, and splitting the analysis into county-level subsets risked underestimating access by excluding clinics outside county borders but closer to tract centroids. To ensure accuracy, this study used ArcGIS StreetMap Premium [35], a commercial geodatabase with comprehensive roadway connectivity, routing attributes, and elevation data. The dataset was integrated into ArcGIS Pro to generate a state-level routing network.

The OD cost matrix was configured to calculate driving time in minutes, using “Driving Time” as the travel mode. The analysis was limited to one destination per origin (the nearest clinic) and generated a “lines” attribute table containing OD travel times and distances. These results were joined to the census tract dataset for mapping and descriptive analysis.

#### 2.4.1. Origin–Destination Cost Matrix

The OD cost matrix tool was used to calculate travel times from the geographic centroid of each census tract to the nearest outpatient clinic, using ArcGIS StreetMap Premium as a routable road network dataset.

#### 2.4.2. Accessibility Index Calculation Using a Two-Step Floating Catchment Area (2SFCA)

The two-step floating catchment area (2SFCA) method, first proposed by Radke and Mu, is a robust approach for assessing healthcare accessibility [36,37,38,39]. Unlike the traditional Gravity method, which is effective when the exact location of the provider is known, the 2SFCA method is a better fit for this study because it effectively measures service accessibility by considering both the proximity of populations to healthcare services and the ratio of providers to population [39,40]. The 2SFCA method involves two primary analytical steps using ‘supply’ points (in this study outpatient clinic locations) and ‘demand’ points (in this study census tract centroids) to compute the accessibility index [41]. Our study adapted the methodology from previous research by Dong et al. and Naylor et al., tailored to the variables of clinic locations and census tracts in Texas [37,38].

Accessibility was quantified using the 2SFCA method, which incorporates both travel time and provider-to-population ratios. Clinic locations served as supply points, and census tract centroids as demand points. A 15 min drive-time threshold was selected as a conservative and clinically meaningful estimate of reasonable travel burden for outpatient rehabilitation, where repeated visits may magnify transportation demands over time [42]. During model development, 30 min catchment maps were also examined and demonstrated broader rural coverage while preserving the overall urban concentration of services, supporting the use of 15 min as the primary analytic threshold.

In Step 1, the provider-to-population ratio was computed within each clinic’s catchment area by aggregating census tract populations falling within a 15 min drive. In Step 2, these ratios were summed for each census tract to produce an accessibility index (AI) that reflects access to outpatient physical and occupational therapy services relative to population demand.

Accessibility index scores were joined to census tract polygons and visualized using graduated color choropleth maps. Census tract polygons represent the border of each census tract and graduated color choropleth maps illustrate the variability in accessibility index across census tracts. This approach captured both clinic density and overlapping service areas, allowing for granular analysis of urban and rural access patterns.

#### 2.4.3. Hot Spot Analysis

A hot spot analysis was conducted in ArcGIS Pro (v3.3, ESRI) to identify statistically significant clusters of high and low accessibility scores derived from the 2SFCA analysis [43]. The Getis-Ord Gi* statistic evaluates spatial autocorrelation by comparing each census tract’s accessibility score to those of its neighbors, producing z-scores and *p*-values that indicate whether clustering occurs beyond random chance. Statistically significant positive z-scores correspond to high-access “hot spots,” while negative z-scores correspond to low-access “cold spots.”

Due to the heterogeneity of Texas census tract sizes, a spatial weights matrix was constructed to ensure realistic neighborhood definitions [44]. A fixed-distance band of 4800 m (three miles) was applied to represent urban spatial relationships, while tracts without a neighbor within this distance were assigned a minimum of two neighbors to accommodate rural geographies. Manhattan distance was used to approximate urban road layouts, yielding an average of eight neighbors per tract.

The Gi* statistic was calculated using ArcGIS Pro’s Hot Spot Analysis tool. A False discovery rate (FDR) correction was applied to control for multiple testing. The output included a categorical Gi_Bin field representing significance at 90%, 95%, and 99% confidence levels, which was mapped to visualize spatial patterns of access. Although the Getis-Ord Gi* statistic yields multiple confidence tiers (90%, 95%, and 99%), these were collapsed into a binary indicator representing statistically meaningful clustering versus no clustering because the analytic aim was to identify the presence of actionable shortage–area clustering rather than differentiate relative levels of statistical certainty.

### 2.5. Statistical Analysis

To examine demographic differences between areas of high and low accessibility, census tracts classified as significant hot or cold spots were extracted from ArcGIS Pro and exported to R (v4.3.1, R Core Team, Vienna, Austria). Because the study objective was to identify demographic differences between statistically significant clusters of high and low accessibility, rather than to model the continuous accessibility surface itself, only census tracts identified as significant Gi* hot or cold spots were included in the regression analysis. The Gi_Bin field was recoded as a binary variable (1 = hot spot; 0 = cold spot) and used as the dependent variable in a stepwise logistic regression model. The predictor variables included: the percent of racial/ethnic groups (Hispanic, White, Black, Asian, and American Indian), the percent of households without vehicles, the percent of single-parent households, the percent of households without internet access, the percent of individuals with disabilities, and the percent of individuals without a high school diploma.

During preliminary model selection, inclusion of both the White percentage and minority percentage resulted in model singularity, indicating linear dependency among racial composition variables; therefore, the minority percentage was excluded from the final model to improve stability and interpretability. The logistic regression model was fitted using the glm() function with a binomial link, and odds ratios (ORs) were calculated to interpret the strength of associations. The model fit was evaluated using null deviance, residual deviance, and Akaike Information Criterion (AIC). Descriptive statistics were generated for all variables, and comparisons between hot and cold spot tracts were reported alongside the regression results. All scripts, code snippets, and model diagnostics are provided in the Supplementary Materials.

## 3. Results

### 3.1. Geospatial Results

#### 3.1.1. Origin–Destination Cost Matrix Results

**Figure 1:**
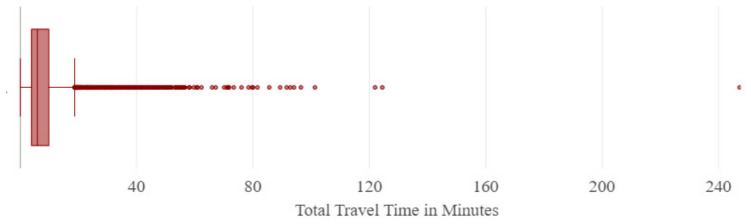
Box plot of distribution of travel time from census tract centroid to nearest source of care.

The OD cost matrix results provide the travel time from each census tract centroid to the nearest source of outpatient rehabilitation facility. Figure 1 is a box plot depicting the distribution of the total driving time from the centroids of census tracts to the nearest outpatient clinic in minutes. The *x*-axis represents the total travel time in minutes, which ranged from 0–240. While most of the data points are clustered around the lower end of travel time and the median value of 20 min, outliers are visible beyond the box plot whiskers, including one extreme value exceeding 240 min that reflects marked rural geographic isolation.

#### 3.1.2. Accessibility Index Results

**Figure 2:**
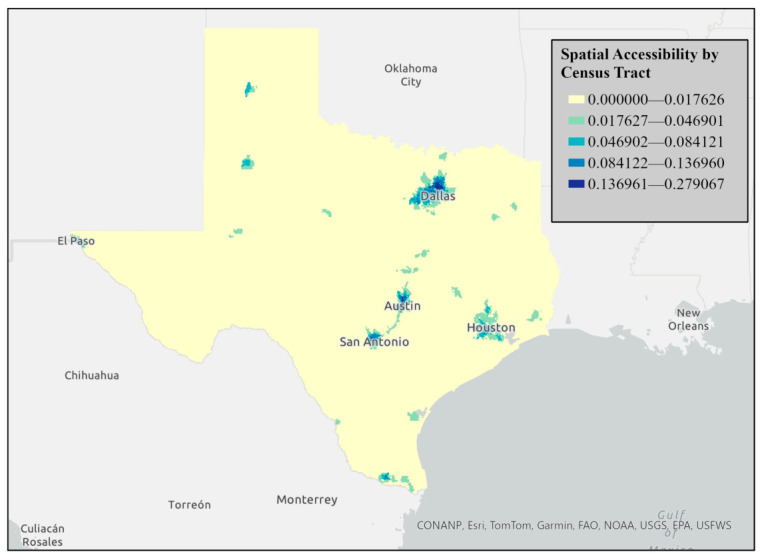
Spatial accessibility by census tract in Texas.

**Figure 3:**
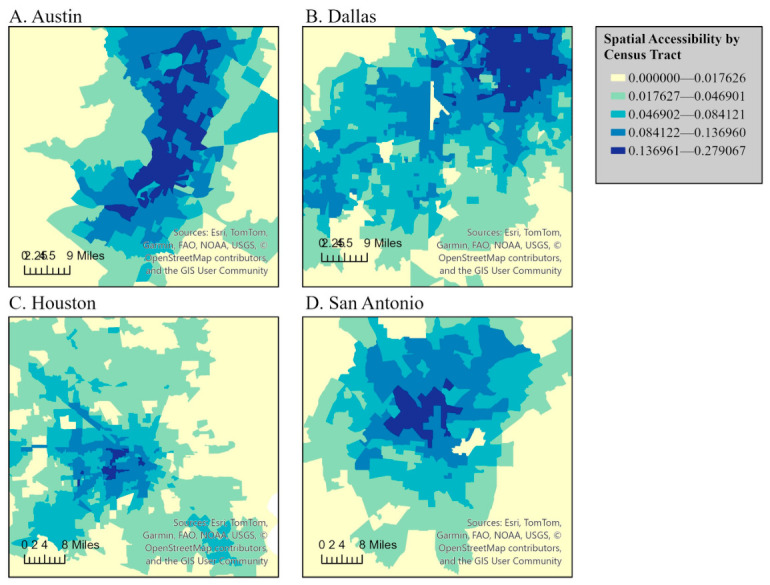
Spatial accessibility by census tract in Texas with views of Austin (**A**), Dallas/Fort Worth (**B**), Houston (**C**), and San Antonio (**D**).

The index of accessibility calculated from the 2SFCA method was visualized using graduated color choropleth maps using natural breaks. Figure 2 and Figure 3 visualize spatial accessibility by the census tract in Texas using a choropleth map based on accessibility index scores calculated through the 2SFCA method. The accessibility index scores are represented with graduated colors. Blue gradients represent accessibility levels; dark blue represents the highest accessibility scores (0.17612–0.277607), medium-to-light blue represents moderate-to-low accessibility scores, and very light blue represents the lowest accessibility (0–0.016726), indicating limited outpatient rehabilitation access.

Figure 2 and Figure 3 demonstrate a clear statewide urban–rural divide in accessibility, with the highest accessibility concentrated in major metropolitan regions and the lowest accessibility across rural West Texas, the Panhandle, and border areas. Within metropolitan regions, higher accessibility was concentrated in urban cores and northern or western suburbs, while lower accessibility was more common in peripheral and historically underserved southern and eastern subregions. These patterns were most pronounced in Dallas–Fort Worth, Houston, and San Antonio, whereas the Austin metropolitan area demonstrated comparatively fewer low-access tracts.

#### 3.1.3. Getis-Ord Gi* Results

**Figure 4:**
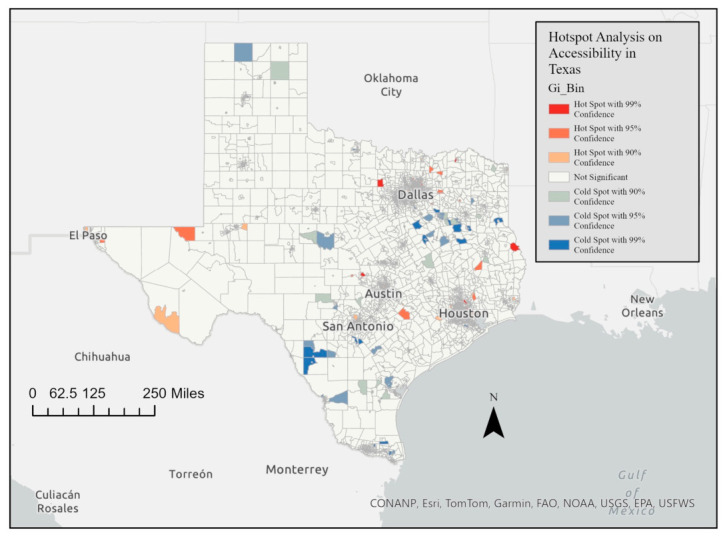
Spatial accessibility index Getis-Ord Gi* statistic in Texas.

**Figure 5:**
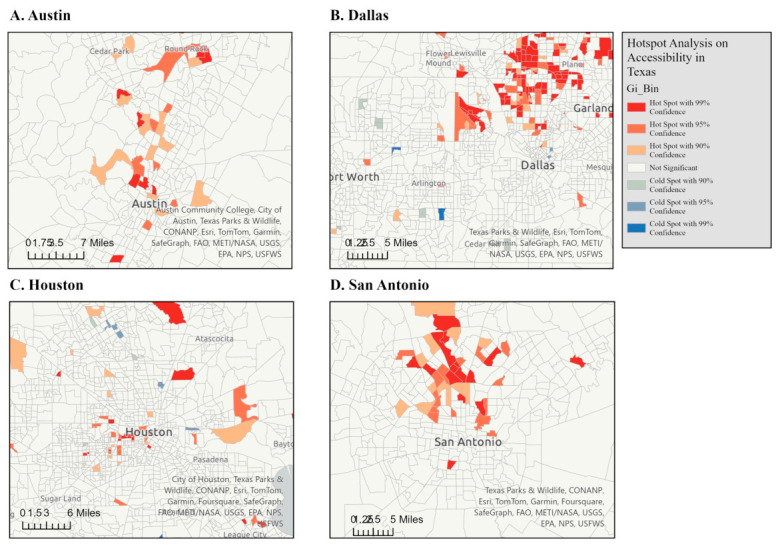
Spatial accessibility index Getis-Ord Gi* statistic in Texas with views of Austin (**A**), Dallas/Fort Worth (**B**), Houston (**C**), and San Antonio (**D**).

The results of the Getis-Ord Gi* statistic are shown in a series of maps illustrating the hot spots and cold spots of the accessibility index across the entire state of Texas. This spatial statistical method identified areas with significantly higher or lower indexes of accessibility. Figure 4 and Figure 5 represent the Getis-Ord Gi* statistic for the spatial accessibility index for Texas (Figure 4), and with zoomed-in views of Austin (A), Dallas/Fort Worth (B), Houston (C), and San Antonio (D). Red areas represent hot spots, where there are statistically significant clusters of high accessibility index scores. Blue areas represent cold spots, where there are statistically significant clusters of low accessibility index scores. In the full state map, the hot spots are concentrated in major metropolitan regions. The cold spots are prevalent in rural areas, particularly in West Texas (e.g., around Midland, Odessa, and areas west of Lubbock), the Texas Panhandle, and the Rio Grande Valley and border regions.

#### 3.1.4. Statistical Results

A stepwise logistic regression analysis was performed to select the most important socioeconomic and demographic predictors that were related to the binary outcome of a location being a hot spot or cold spot. The binary outcome of hot spot versus cold spot was determined by converting the categorical variable ‘Gi_Bin’ into a binary variable. A Gi_Bin of three, two or one, representative of a hot spot of 99, 95, and 90% confidence intervals for increased accessibility was converted to the binary outcome “1.” A Gi_Bin of −3, −2, or −1 representative of a cold spot was converted to the binary outcome of “0” (Table 1). The model demonstrated a significant improvement in fit compared to the null model (AIC = 527.16), with a residual deviance of 371.64 (AIC = 395.64), indicating that the included predictors meaningfully explain the variation in the outcome.

Several predictors were statistically significant (*p* < 0.05). Percent Hispanic (β = 0.136, OR = 1.146, and *p* < 0.001), percent White (β = 0.107, OR = 1.113, and *p* = 0.009), and percent Black (β = 0.099, OR = 1.104, and *p* = 0.015) were all positively associated with an increased odds of being in a hot spot. Similarly, percent Asian (β = 0.111, OR = 1.117, and *p* = 0.008) and percent households with single parents (β = 0.124, OR = 1.132, and *p* < 0.001) showed positive relationships with the outcome. Additionally, percent with disability had a positive effect (β = 0.170, OR = 1.185, and *p* < 0.001), suggesting that higher percentages of individuals with disabilities significantly increase the odds of a being in a hot spot.

Conversely, percent no vehicle households (β = −0.075, OR = 0.928, and *p* = 0.004), percent households with no partner (β = −0.045, OR = 0.956, and *p* < 0.001), and percent with no high school diploma (β = −0.073, OR = 0.930, and *p* < 0.001) were negatively associated with the outcome. These results suggest that areas with higher rates of households lacking vehicles, single individuals without partners, and individuals without high school diplomas are associated with reduced odds of being in a hot spot with increased accessibility.

## 4. Discussion

This statewide geospatial analysis identified substantial disparities in outpatient rehabilitation accessibility across Texas. The accessibility index derived from the 2SFCA showed consistently higher access in the major metropolitan corridors (Dallas–Fort Worth, Houston, Austin, San Antonio) and lower access in rural West Texas, the Panhandle, and border regions. Within cities, accessibility was uneven, with pockets of lower access on urban peripheries and in historically under-resourced neighborhoods. Hot spot analysis (Getis-Ord Gi*) corroborated these patterns, locating statistically significant clusters of high access in urban areas and low access across large rural areas. OD cost matrix results reinforced the accessibility gradient: most urban census tracts were within relatively short drive times of a clinic, whereas rural tracts had markedly longer travel times, sometimes exceeding practical thresholds for multi-visit rehabilitation. For individuals living with a disability who require frequent, ongoing therapy, extended travel burdens may translate directly into missed care, delayed recovery, and reduced functional participation.

### 4.1. The Origin–Destination Cost Matrix

The OD cost matrix results showed a median nearest-clinic travel time of 20 min statewide, while most metropolitan census tracts were located within approximately 30 min of outpatient rehabilitation care. In contrast, rural residents experienced substantially longer travel burdens, with some trips exceeding two hours. However, rural residents face significant travel burdens, with some trips exceeding two hours. This access disparity underscores geographic inequities and the need for targeted interventions to increase rehabilitation access in underserved regions. Even for urban residents, frequent rehabilitation appointments, often two or three times a week, can accumulate substantial travel time, adding up to nearly three hours per week even when the commute is only 30 min away. Evidence on patients’ willingness to travel for rehabilitation services is limited, though some studies suggest that patients are reluctant to travel more than fifteen minutes for care [42], pointing to the importance of considering travel burden in planning service delivery. This suggests that more research is needed to understand what would be considered a reasonable travel time for individuals undergoing rehabilitation care.

### 4.2. Accessibility Index

The 2SFCA analysis highlights striking disparities in rehabilitation accessibility across Texas. Urban centers such as Austin, Dallas, Houston, and San Antonio demonstrate higher accessibility due to dense clinic networks and major transportation corridors, whereas rural areas, including West Texas, the Panhandle, and border regions, face significant challenges, reflecting longstanding urban–rural divides in healthcare access [45,46,47]. Affluent suburbs in northern Dallas and San Antonio, and western Houston, show particularly high accessibility, while historically underserved neighborhoods in South Dallas, South San Antonio, and East Houston remain low-access [48,49,50]. These patterns emphasize systemic inequities linked to both socioeconomic and geographic factors. Florida and Mellander completed a large study on segregation within metropolitan regions in the United States [51]. Austin (first), San Antonio (third), Houston (fourth) and Dallas–Fort Worth–Arlington, TX (seventh) encompassed four of the highest seven economically segregated metropolitan regions within the United States. Prior research points to Texas cities being economically segregated, or having wealth concentrated in specific regions resulting in neighborhood divisions [50]. This study was the first to demonstrate that rehabilitation accessibility follows similar patterns to economic segregation.

### 4.3. Implications for Access Interventions

The disparities identified in this study carry implications for equitable rehabilitation access. Equitable rehabilitation access means ensuring that everyone has fair and just opportunities to receive rehabilitation services they need to achieve their full potential, regardless of their background, identity, circumstances or geography. Tract-level accessibility maps offer a tool for health systems and policymakers to guide service expansion and prioritize cold-spot areas, and they can inform workforce distribution. Strategies include developing additional outpatient rehabilitation clinics, introducing mobile rehabilitation clinics, and expanding telehealth rehabilitation capacity and reimbursement to reach remote communities. Evidence suggests that mobile health clinics can deliver cost-effective care for underserved populations [52,53,54]. Implementing financial incentive programs for rehabilitation providers to establish or expand practices in regions with reduced accessibility can enhance equity by addressing workforce shortages, as financial incentives have been shown to improve provider distribution in underserved areas [55,56]. The findings provide a practical framework for generating hypotheses about place-based intervention priorities based on the specific spatial and demographic disparities identified in this study. For example, persistent cold spots in rural West Texas, the Panhandle, and border regions may represent important settings for future evaluation of strategies such as workforce incentive programs, mobile outpatient rehabilitation services, and tele-rehabilitation expansion. Within metropolitan regions, lower-access areas identified in South Dallas, East Houston, and southern San Antonio may similarly warrant further investigation of targeted clinic placement, transportation partnerships, and transit-oriented service expansion as potential approaches to improving access.

### 4.4. Transportation Networks and Access

Transportation infrastructure plays a central role in shaping healthcare access. Areas along major highways, such as Interstate 35 in Austin and key corridors in Dallas and Houston, show higher accessibility, emphasizing the influence of roadway connectivity. This finding underscores the importance of transportation infrastructure in ensuring equitable healthcare access and highlights the need for integrated solutions that address both healthcare distribution and mobility challenges. These driving time disparities may be further compounded in metropolitan regions where public transit options are limited, particularly for households without vehicles. Although public transit was not directly modeled in this analysis, these findings suggest that transportation constraints beyond roadway travel may further intensify access inequities in already low-access areas [57,58,59].

### 4.5. Getis-Ord Gi* Statistic

Hot spot analysis using the Getis-Ord Gi* statistic revealed clear clustering patterns: high-access tracts are concentrated in major metropolitan centers, while large regions of West Texas, the Panhandle, and the Rio Grande Valley remain persistently low-access. Within metropolitan areas, cold spots appear in peripheral or historically underserved neighborhoods, though these disparities are less pronounced relative to rural gaps. This clustering reinforces the urban–rural divide and highlights areas for targeted resource allocation.

### 4.6. Population Characteristics

Logistic regression analysis provided insight into demographic and socioeconomic predictors of access. Tracts with higher proportions of Hispanic, White, Black, and Asian residents were positively associated with high-accessibility hot spots, reflecting patterns of metropolitan diversity and provider clustering [60,61]. Conversely, households without vehicles and households without partners were strongly associated with low-access areas, underscoring the compounding effects of transportation barriers on rehabilitation access inequities [62,63].

Interestingly, single-parent households were positively associated with hot spot presence, while no-partner households were significantly negatively associated, a surprising finding given the established links between single-parent status and economic vulnerability [64,65,66,67]. This complexity highlights the need for further research into local-level dynamics. Disability prevalence was also positively associated with access, which may reflect the metropolitan co-location of larger populations, higher disability prevalence, and denser outpatient clinic networks, rather than implying that higher disability prevalence directly drives clinic placement. Lower educational attainment strongly correlated with a low access, consistent with evidence that individuals with less education experience greater barriers to care, poorer health outcomes, and lower insurance coverage rates [68,69]. Digital access disparities, measured by household internet connectivity, showed only marginal significance, but this trend underscores the role of technology in modern care delivery.

### 4.7. Strengths

This study offers a statewide, granular assessment of outpatient rehabilitation access, analyzing 6896 census tracts and 2255 verified PT/OT clinic sites. We combined three complementary geospatial approaches (OD travel time, 2SFCA-derived accessibility index, and Getis-Ord Gi* clustering) to characterize both continuous access gradients and statistically significant clusters of high and low accessibility. A documented, auditable cleaning protocol (standardization, de-duplication, and web-based verification) improves confidence in clinic location accuracy. Use of StreetMap Premium enabled routable, elevation-aware travel time estimation across urban and rural Texas, overcoming limitations of open datasets and avoiding county-by-county splits that can misidentify the nearest clinic. The tract-level integration of ACS demographics supports equity-oriented interpretation and future policy intervention as well as the potential to act locally within areas of metropolitan centers to improve accessibility in areas of most need.

### 4.8. Limitations

These findings are derived from cross-sectional data and may not capture recent openings/closures or population shifts (clinics: 2022; ACS: 2020). Licensure-based addresses, though cleaned and verified, can contain residual misclassification (e.g., administrative offices and multi-suite sites). Although address verification followed a standardized cleaning workflow, the manual reconciliation of duplicate practice sites may introduce some subjectivity, particularly for multi-suite facilities, shared buildings, or health system naming variations. The OD analysis uses centroids and nearest clinic driving time; it does not account for patient choice, clinic capacity, payer networks, waiting times, parking/transit barriers, or tele-rehabilitation. Census tract centroids were used as demand points, consistent with common 2SFCA and origin–destination network approaches using areal census data. However, geometric centroids may introduce spatial representation error, particularly in large rural tracts where the centroid may not align with actual population settlement patterns. As a result, travel times in sparsely populated regions may be over- or underestimated. Future studies may improve precision by incorporating population-weighted centroids or asymmetric population surfaces. Hot spot detection required a fixed-distance band (4800 m) with a minimum-neighbor rule and a Manhattan metric for heterogeneous tract sizes; these decisions balance urban realism and rural inclusion but may under- or over-connect some sparsely populated areas. Dichotomizing the Gi_Bin variable into hot versus cold spots simplified interpretation and facilitated direct comparison of high- and low-access regions; however, this approach may reduce the granularity of spatial clustering intensity. In addition, comparisons between urban and rural census tracts may be influenced by the modifiable areal unit problem, as tract size and population density vary substantially across Texas. These differences in areal scale may affect the apparent magnitude and spatial concentration of accessibility patterns. Finally, the analysis focuses on outpatient PT/OT only; other rehabilitation settings (e.g., outpatient speech therapy, inpatient rehabilitation centers, and home health services), as well as clinic capacity and quality considerations, were beyond the scope of this study and including these settings may change the broader access picture.

### 4.9. Future Directions

Future research should focus on regional hot spot analysis within metropolitan areas to better capture neighborhood-level disparities. More advanced techniques could be considered within metropolitan analyses, such as dynamic spatial weighting or temporal analyses [70,71]. These methods would allow for the incorporation of longitudinal data to examine how changes in clinics’ availability, infrastructure, or policy interventions impact spatial accessibility over time. Finally, the inclusion of additional rehabilitation services, such as speech therapy or more comprehensive occupational and physical therapy practice settings could provide a more comprehensive view of healthcare access disparities.

## 5. Conclusions

This study highlights significant spatial disparities in access to outpatient physical therapy and occupational therapy rehabilitation services across Texas, with pronounced differences between urban and rural regions and within metropolitan areas. The study utilized advanced spatial analysis methods including the 2SFCA and Getis-Ord Gi* statistics to provide insights into hot spots of high accessibility and cold spots of underserved areas. The findings underscore the importance of targeted interventions, such as clinic placement and improved transportation, to address inequities. In the future, a focus on localized analyses and incorporating longitudinal data could provide better information for healthcare policy and planning.

## Figures and Tables

**Table 1:** Logistic regression results predicting hot spot.

Variable	Unstandardized β	SE	z-Value	*p*-Value	Odds Ratio	CI (Lower)	CI (Upper)
Intercept	−9.34	3.98	−2.35	0.019	8.79 × 10^−5^	2.59 × 10^−8^	0.193
Hispanic	0.136	0.041	3.29	<0.001	1.146	1.058	1.246
White	0.107	0.041	2.62	0.009	1.113	1.029	1.211
Black	0.099	0.041	2.43	0.015	1.104	1.020	1.200
American Indian	0.371	0.257	1.44	0.149	1.449	0.901	2.483
Asian	0.111	0.042	2.65	0.008	1.117	1.030	1.216
No Vehicle Households	−0.075	0.026	−2.88	0.004	0.928	0.880	0.975
Households No Partner Present	−0.045	0.011	−4.12	<0.001	0.956	0.934	0.975
Households Single Parent	0.124	0.027	4.5	<0.001	1.132	1.07	1.196
No Internet Households	0.037	0.019	1.94	0.052	1.038	1.001	1.078
With Disability	0.17	0.031	5.51	<0.001	1.185	1.118	1.262
No HS Diploma	−0.073	0.019	−3.75	<0.001	0.930	0.894	0.964

(1) Abbreviations: SE, standard error; β, log-odds unstandardized coefficient. (2) All predictors refer to the percentage of the population in the census tract.

## Data Availability

The data that support the findings of this study are available from publicly available datasets included in this published study and through the Executive Council of Physical Therapy and Occupational Therapy Examiners, but restrictions apply to the availability of these data, which were used under license for the current study, and so are not publicly available. Data are, however, available from the authors upon reasonable request and with permission of the Executive Council of Physical Therapy and Occupational Therapy Examiners.

## Supplementary Materials

The following supporting information can be downloaded at: https://www.mdpi.com/article/10.3390/ijerph23040517/s1. Table S1: Data Sources; Table S2: Summary of clinic address verification and duplicates removed; Table S3: 2SFCA model equation key; Table S4: Step-by-step methods for conducting 2SFCA calculation using ArcGIS Pro [58]; Table S5: Logistic regression output (coefficients on log-odds scale); Table S6: Model selection comparison (AIC); Figure S1: Workflow for geospatial and data analysis; Figure S2: Data cleaning and verification process of outpatient clinic locations in Texas; Figure S3. This scatterplot illustrates the near-perfect inverse relationship between tract-level percent White and percent minority composition. As expected, increases in percent White correspond directly to decreases in percent minority, producing an essentially linear dependency between the variables. This relationship helps explain the singularity encountered when both variables were considered simultaneously in the regression model.

## Abbreviations

The following abbreviations are used in this manuscript:

2SFCA	Two-step floating catchment area
AI	Accessibility Index
ACS	American Community Survey
OD	Origin–destination
Gi*	Getis-Ord statistic
PT	Physical therapy
OT	Occupational therapy
FDR	False discovery rate

## References

[1] County Health Rankings & Roadmaps Access to Care. https://www.countyhealthrankings.org/.

[2] Graham Center Access Denied. https://www.graham-center.org/content/dam/rgc/documents/publications-reports/monographs-books/Access%20Denied.pdf.

[3] Kaiser Family Foundation The Uninsured: A Primer. https://files.kff.org/attachment/primer-the-uninsured-a-primer-key-facts-about-health-insurance-and-the-uninsured-in-the-era-of-health-reform.

[4] Penchansky R., Thomas J.W. (1981). The concept of access: Definition and relationship to consumer satisfaction. Med. Care.

[5] Saurman E. (2016). Improving access: Modifying Penchansky and Thomas’s theory of access. J. Health Serv. Res. Policy.

[6] American Physical Therapy Association Direct Access Advocacy. https://www.apta.org/advocacy/issues/direct-access-advocacy.

[7] Clark B., Clark L., Showalter C., Stoner T. (2022). A call to action: Direct access to physical therapy is highly successful in the US military. When will professional bodies, legislatures, and payors provide the same advantages to all US civilian physical therapists?. J. Man. Manip. Ther..

[8] Zahnd W.E., Del Vecchio N., Askelson N., Eberth J.M., Vanderpool R.C., Overholser L., Madhivana P., Hirschey R., Edward J. (2022). Definition and categorization of rural and assessment of realized access to care. Health Serv. Res..

[9] Khan A.A., Bhardwaj S.M. (1994). Access to health care: A conceptual framework and its relevance to health care planning. Eval. Health Prof..

[10] Sullivan J., Orris A., Lukens G. Entering Their Second Decade, Affordable Care Act Coverage Expansions Have Helped Millions, Provide the Basis for Further Progress. https://www.cbpp.org/research/health/entering-their-second-decade-affordable-care-act-coverage-expansions-have-helped.

[11] U.S. Government Accountability Office Why Health Care Is Harder to Access in Rural America. https://www.gao.gov/blog/why-health-care-harder-access-rural-america.

[12] Bodenheimer T., Grumbach K. (2020). Understanding Health Policy: A Clinical Approach.

[13] Wong A., Hoang T.M.H., Ferrara V., Nguyen T.H. (2025). How systemic barriers can impact health inequities when facing climate change stressors: A scoping review of global differences. GeoHealth.

[14] Arrington J.C. One Big Beautiful Bill Act. https://www.congress.gov/bill/119th-congress/house-bill/1.

[15] Johns Hopkins Bloomberg School of Public Health The Changes Coming to the ACA, Medicaid, and Medicare. https://publichealth.jhu.edu/2025/the-changes-coming-to-the-aca-medicaid-and-medicare.

[16] American Physical Therapy Association Health Policy & Administration PTJ-PAL Vol. 19 No. 3. https://www.aptaali.org/news/440888/Read-the-February-2019-PTJ-PAL.htm.

[17] Gao F., Foster M., Liu Y. (2019). Disability concentration and access to rehabilitation services: A pilot spatial assessment applying geographic information system analysis. Disabil. Rehabil..

[18] Lakhani A., Parekh S., Gudes O., Grimbeek P., Harre P., Stocker J., Kendall E. (2019). Disability support services in Queensland, Australia: Identifying service gaps through spatial analysis. Appl. Geogr..

[19] Kinder Institute for Urban Research After Census Redefines Urban and Rural, Texas Remains Steadfastly Both. https://kinder.rice.edu/urbanedge/census-redefines-urban-rural.

[20] Mora D.S., Noble G., Alamos C.L., McKinley Sandoval Harding B.M., Moore T.P.H. Three Rural Definitions. https://www.ers.usda.gov/media/5283/texas.pdf.

[21] Rural Health Information Hub Transportation to Support Rural Healthcare. https://www.ruralhealthinfo.org/topics/transportation.

[22] Wolfe M.K., McDonald N.C., Holmes G.M. (2020). Transportation barriers to health care in the United States: Findings from the National Health Interview Survey, 1997–2017. Am. J. Public Health.

[23] Jiao J. (2017). Identifying transit deserts in major Texas cities where the supplies missed the demands. J. Transp. Land Use.

[24] Texas Department of Transportation 2023 Texas Transit Statistics. https://www.txdot.gov/content/dam/docs/division/gov/texas-transit-statistics-report-2023..pdf.

[25] Autoinsurance.com Commuting in the U.S. Facts and Statistics. https://www.autoinsurance.com/research/us-commuting-statistics/.

[26] Centers for Disease Control and Prevention Disability & Health U.S. State Profile Data: Texas. https://dhds.cdc.gov/SP?LocationId=48&CategoryId=DISEST&ShowFootnotes=true&showMode=&IndicatorIds=STATTYPE,AGEIND,SEXIND,RACEIND,VETIND&pnl0=Chart,false,YR7,CAT1,BO1,,,,AGEADJPREV&pnl1=Chart,false,YR7,DISSTAT,,,,,PREV&pnl2=Chart,false,YR7,DISSTAT,,,,,AGEADJPREV&pnl3=Chart,false,YR7,DISSTAT,,,,,AGEADJPREV&pnl4=Chart,false,YR7,DISSTAT,,,,,AGEADJPREV&t=1776392452850.

[27] Ratoza M.R., Patel R.M., Brewer W., Mitchell K., Chevan J. (2026). Geographic disparities in rehabilitation provider availability and community demographics in Texas: A cross-sectional geographic information systems study. Phys. Ther..

[28] Texas Department of State Health Services. https://www.dshs.texas.gov/.

[29] U.S. Census Bureau American Community Survey (ACS). https://www.census.gov/programs-surveys/acs.

[30] Texas Executive Council of Physical Therapy and Occupational Therapy Examiners Open Records Request. https://ptot.texas.gov/open-records-request/.

[31] U.S. Census Bureau Census.gov. https://www.census.gov/.

[32] Ratoza M., Patel R.M., Chevan J., Brewer W., Mitchell K. (2025). Mapping the availability of rehabilitation providers using public licensure and population data for a geographic information system-based approach to workforce planning: Cross-sectional feasibility study. JMIR Form. Res..

[33] Esri ArcGIS. https://www.esri.com/en-us/arcgis/geospatial-platform/overview.

[34] Esri ArcGIS World Geocoding. https://www.arcgis.com/sharing/rest/content/items/305f2e55e67f4389bef269669fc2e284.

[35] Esri On-Premises and Offline Routing, Geocoding & Mapping. https://www.esri.com/en-us/arcgis/products/arcgis-streetmap-premium/overview.

[36] Radke J., Mu L. (2000). Spatial decompositions, modeling and mapping service regions to predict access to social programs. Ann. GIS.

[37] Dong Y., Fu L., Tan R., Ding L. (2019). The dilemma of medical reimbursement policy in rural China: Spatial variability between reimbursement region and medical catchment area. Int. J. Environ. Res. Public Health.

[38] Naylor K.B., Tootoo J., Yakusheva O., Shipman S.A., Bynum J.P.W., Davis M.A. (2019). Geographic variation in spatial accessibility of U.S. healthcare providers. PLoS ONE.

[39] Pervorse A. Spatial Accessibility in Health Care. https://storymaps.arcgis.com/stories/afec06281a264b3e9bdcb02b1ece8778.

[40] Gautam S., Li Y., Johnson T.G. (2014). Do alternative spatial healthcare access measures tell the same story?. GeoJournal.

[41] Kanuganti S., Sarkar A.K., Singh A.P. (2016). Quantifying accessibility to health care using two-step floating catchment area method (2SFCA): A case study in Rajasthan. Transp. Res. Procedia.

[42] Cypress Health Partners Why Distance Is So Important for Patient Access, and What We’re Doing About It. https://cypresshealthpartners.com/why-distance-is-so-important-for-patient-access-and-what-were-doing-about-it/.

[43] Esri How Hot Spot Analysis (Getis-Ord Gi*) Works. https://pro.arcgis.com/en/pro-app/latest/tool-reference/spatial-statistics/h-how-hot-spot-analysis-getis-ord-gi-spatial-stati.htm.

[44] Grekousis G. (2020). Spatial Analysis Methods and Practice: Describe—Explore—Explain Through GIS.

[45] Chen X., Orom H., Hay J.L., Waters E.A., Schofield E., Li Y., Kiviniemi M.T. (2019). Differences in rural and urban health information access and use. J. Rural Health.

[46] McManus B.M., Lindrooth R., Richardson Z., Rapport M.J. (2016). Urban/rural differences in therapy service use among Medicaid children aged 0–3 with developmental conditions in Colorado. Acad. Pediatr..

[47] Quigley D.D., Chastain A.M., Kang J.A., Bronstein D., Dick A.W., Stone P.W., Shang J. (2022). Systematic review of rural and urban differences in care provided by home health agencies in the United States. J. Am. Med. Dir. Assoc..

[48] Young S. Dallas County Remains Segregated by Race, Income and Education Level, New Study Says. https://www.dallasobserver.com/news/dallas-county-has-widespread-ethnic-disparities-according-to-new-study-10566546.

[49] University of Richmond Mapping Inequality. https://dsl.richmond.edu/panorama/redlining/.

[50] Munson L. What Is Economic Segregation, and Why Does It Matter?. https://hebfdn.org/echoes/economic-segregation-matter/.

[51] Florida R., Mellander C. Segregated City: The Geography of Economic Segregation in America’s Metros. https://hj.diva-portal.org/smash/get/diva2:868382/FULLTEXT01.

[52] Yu S.W.Y., Hill C., Ricks M.L., Bennet J., Oriol N.E. (2017). The scope and impact of mobile health clinics in the United States: A literature review. Int. J. Equity Health.

[53] Malone N.C., Williams M.M., Smith Fawzi M.C., Bennet J., Hill C., Katz J.N., Oriol N.E. (2020). Mobile health clinics in the United States. Int. J. Equity Health.

[54] Tulane University School of Public Health How Do Mobile Health Clinics Improve Access to Health Care?. https://publichealth.tulane.edu/blog/mobile-health-clinics/.

[55] Bärnighausen T., Bloom D.E. (2009). Financial incentives for return of service in underserved areas: A systematic review. BMC Health Serv. Res..

[56] University of Washington Center for Health Workforce Studies State Incentive Programs that Encourage Allied Health Professionals to Provide Care for Underserved Populations. https://familymedicine.uw.edu/chws/studies/state-incentive-programs-that-encourage-allied-health-professionals-to-provide-care-for-underserved-populations/.

[57] Health Affairs Public Transportation in the US: A Driver of Health and Equity. https://www.healthaffairs.org/do/10.1377/hpb20210630.810356/full/.

[58] Centers for Disease Control and Prevention Public Transportation System: Introduction or Expansion. https://archive.cdc.gov/www_cdc_gov/policy/hi5/publictransportation/index.html.

[59] Smith L.B., Yang Z. Public Transportation Facilitates Access to Health Care, Particularly for People Covered by Medicaid. https://www.urban.org/urban-wire/public-transportation-facilitates-access-health-care-particularly-people-covered-medicaid.

[60] Kopczewska K., Kubara M., Kopyt M. (2024). Population density as the attractor of business to the place. Sci. Rep..

[61] Combes P.P., Gobillon L. (2015). The empirics of agglomeration economies. Handbook of Regional and Urban Economics.

[62] Cochran A.L., McDonald N.C., Prunkl L., Vinella-Brusher E., Wang J., Oluyede L., Wolfe M. (2022). Transportation barriers to care among frequent health care users during the COVID pandemic. BMC Public Health.

[63] Syed S.T., Gerber B.S., Sharp L.K. (2013). Traveling towards disease: Transportation barriers to health care access. J. Community Health.

[64] The Annie E. Casey Foundation Child Well-Being in Single-Parent Families. https://www.aecf.org/blog/child-well-being-in-single-parent-families.

[65] Esri Where Are Children in Single-Parent Families?. https://www.arcgis.com/home/item.html?id=2a15d985d0ad466ab8532885a07ae61d.

[66] Robinson M. Map Reveals States with Most Single-Parent Households. https://www.newsweek.com/us-census-data-map-reveals-states-most-single-parent-households-1908428.

[67] Kent D.U.S. Has World’s Highest Rate of Children Living in Single-Parent Households. https://www.pewresearch.org/short-reads/2019/12/12/u-s-children-more-likely-than-children-in-other-countries-to-live-with-just-one-parent/.

[68] Raghupathi V., Raghupathi W. (2020). The influence of education on health: An empirical assessment of OECD countries for the period 1995–2015. Arch. Public Health.

[69] Sullivan K. Lack of Education Breeds Disparities Despite Equal Access to Care. https://www.fiercehealthcare.com/healthcare/lack-education-breeds-disparities-despite-equal-access-to-care.

[70] Esri Temporal Analysis. https://doc.arcgis.com/en/insights/latest/analyze/temporal-analysis.htm.

[71] Janatabadi F., Ermagun A. (2024). Access weight matrix: A place and mobility infused spatial weight matrix. Geogr. Anal..

