# Supplementary material for: Geospatial Disparities in Access to Outpatient Physical and Occupational Therapy Services in Texas: Implications for Health Equity and Rehabilitation Workforce Policy
Source: Int J Environ Res Public Health. 2026 Apr 17;23(4):517. doi: 10.3390/ijerph23040517 (PMC13115661; doi:10.3390/ijerph23040517)
Supplement: Supplementary file 1 [file ijerph-23-00517-s001.zip › ijerph-4222128-supplementary.pdf]

Supplementary Material

1. Data Sources and Cleaning

1.1 Data Sources

Table S1. Data Sources

Dataset	Year(s) of Data	Extraction Date	Description of Variables	Notes
Outpatient Clinic Addresses	2022	Sept 2023	Clinic addresses for all licensed PT and OT practice sites in Texas.	Extracted from THHS; cleaned to include outpatient, patient-facing sites only.
Therapist Residential Addresses	2022	Nov 2022	Residential addresses of all actively licensed PTs and OTs.	Obtained via ECPTOTE open records request; used for workforce mapping.
Community-Level Demographics	2020	Nov 2023	Census tract data: population, race/ethnicity, poverty, disability, vehicle access, education.	From ACS 5-Year Estimates.

1.2 Data Cleaning Workflow

Table S2. Summary of clinic address verification and duplicates removed.

Step	Description	Count (n)
Initial combined dataset	All licensed PT/OT practice sites in Texas (from THHS licensure database, Sept 2022)	21,634
Exact duplicates removed	Identified with Excel conditional formatting & COUNTIF	3,090

Near duplicates removed	Standardized via manual review	11,104
Addresses verified	Verified via Google search & phone calls (pilot-tested, reviewed by second researcher)	All remaining
Additional duplicates removed during verification	Identified and removed during manual verification	332
Final outpatient clinic count	Unique, verified outpatient PT/OT practice sites included in analysis	2,255

Note: Figure S1 provides a visual flowchart of this data cleaning and verification process.

Figure S1. Workflow for Geospatial and Data Analysis

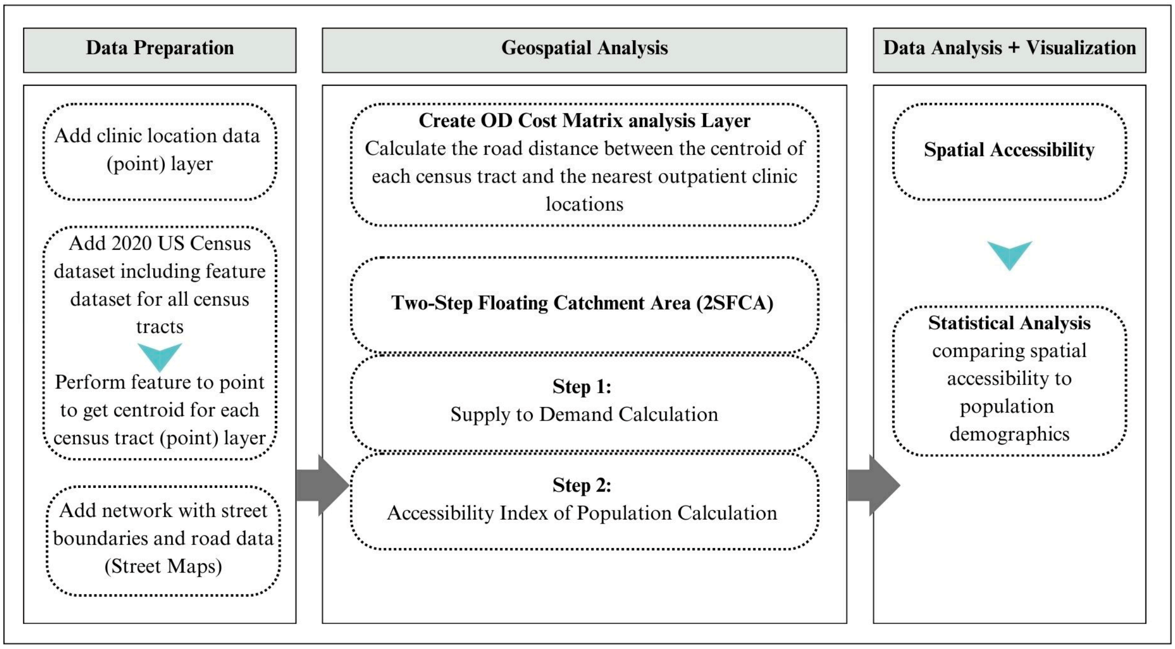

Figure S2. Data Cleaning and Verification Process of Outpatient Clinic Locations in Texas

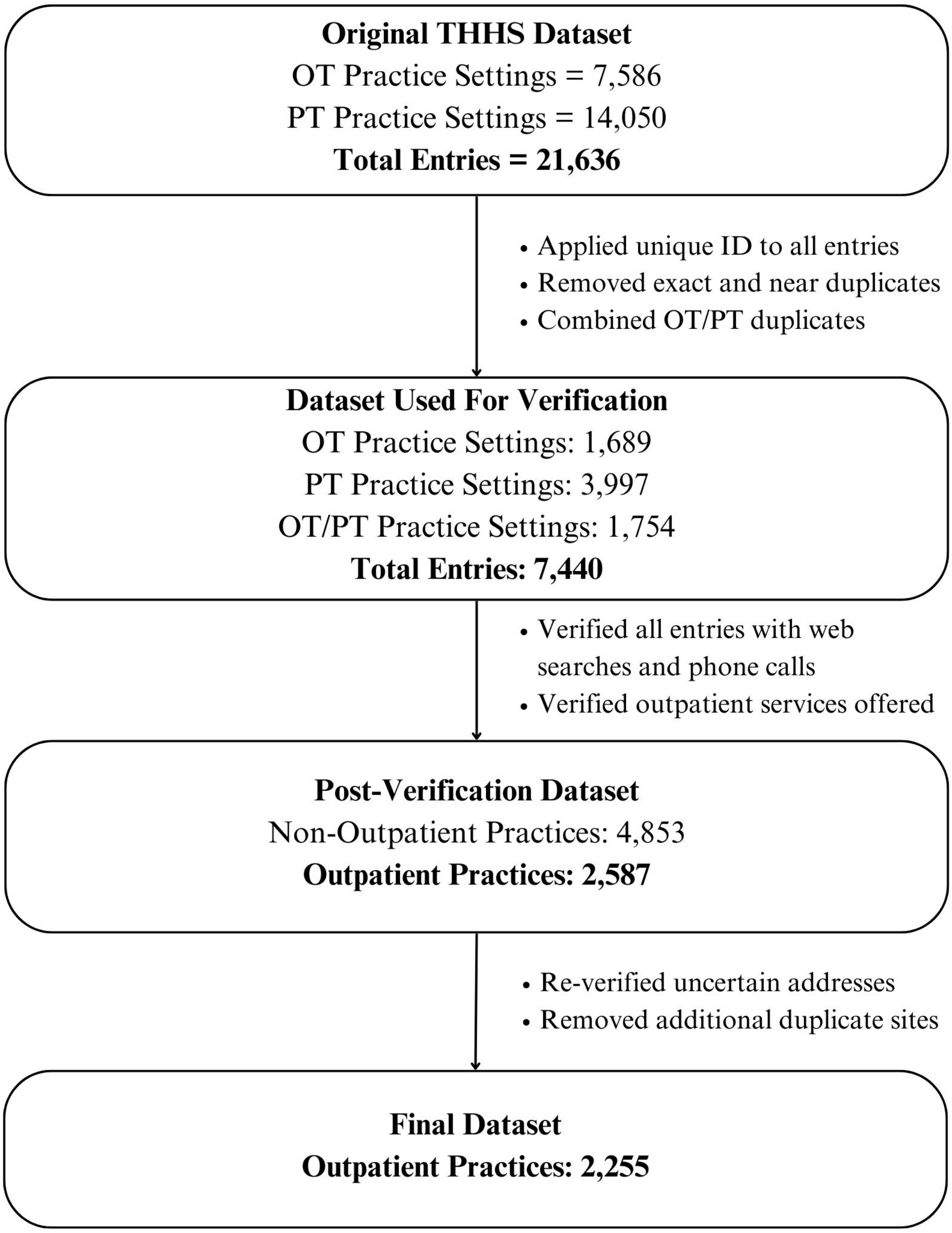

2. GIS Workflow Documentation

2.1 Origin–Destination (OD) Cost Matrix

An Origin–Destination (OD) Cost Matrix analysis was performed in ArcGIS Pro (v3.3, ESRI) to calculate travel times from the geographic centroid of each Texas census tract (n = 6,896) to the nearest outpatient rehabilitation clinic (n = 2,255). Centroids were created using the Feature to Point tool, with each tract represented as a single origin point. StreetMap Premium, a commercial dataset with comprehensive road connectivity, routing, and elevation attributes, was used to ensure accurate statewide travel-time modeling. The OD Cost Matrix tool was configured with Driving Time as the travel mode, limiting results to one destination per origin. The resulting output table included origin-to-destination travel time and distance in minutes, which was later joined to census tract attributes for mapping and descriptive summaries.

2.2 Two-Step Floating Catchment Area (2SFCA)

The 2SFCA method was used to calculate a tract-level Accessibility Index incorporating both provider supply and population demand. A 15-minute drive-time catchment threshold was selected based on prior literature demonstrating this interval as a reasonable distance for outpatient service access in urban and rural contexts. The analysis proceeded in two steps:

1. Supply Ratio Calculation: For each clinic (supply point), a 15-minute catchment was generated, and the provider-to-population ratio (Rj) was calculated based on all census tracts (demand points) within that catchment.
2. Accessibility Index Calculation: For each tract, the provider-to-population ratios from all clinics within 15 minutes were summed, yielding the final Accessibility Index (Ai).

Mathematical definitions of variables and equations are provided below:

$$R_j = \frac{S_j}{\sum_{\in\{d\_kj \leq d\_0\}} \quad D_k}$$

$$A_i = \sum_{j\in\{d_{ij} \leq d_0\}} R_j$$

Table S3: 2SFCA Model Equation Key

$\mathbf{D_k}$	The population (Demand) at location k whose centroid falls within the catchment ($d_{kj} \leq d_o$)
$\mathbf{S_j}$	The provider count (Supply) within catchment area
$\mathbf{d_{kj}}$	The travel distance between k and j
$\mathbf{A_i}$	The accessibility of population at a given location i to healthcare based on 2SFCA method
$\mathbf{R_j}$	Healthcare to population ratio at location j whose centroid falls within the catchment ($d_{ij} \leq d_0$)

**d_{ij}**	The travel distance between i and j

Table S4: Step-by-step methods for conducting 2SFCA calculation using ArcGIS Pro[58]

**Step 1: Supply (S) to demand (D) calculation**		
Action	Instructions	Output
Run the first catchment area from each clinic location	Steps: Analysis Tab > Network Analysis > Service Area > Import Facilities > Set Driving Time to “away from facilities” and “15 minutes,” > Settings to “generalized,” “overlap, > Click	Generates a polygon feature layer
Run spatial join to join first catchment area with all census tract centroids within 15-minute drive time	Analysis > Geoprocessing > Spatial Join > Set Target Feature to the Feature Polygon Layer generated from first catchment area, Join Feature to the census tract centroids, choose “Join one to one,” and “keep all target features,” set match option to “intersect,” hover over field “provider count” and click “edit,” and then click “sum” > Click Run	A new feature layer that has joined the first catchment area to centroid data layer
Calculate provider to population field	Open the attribute table for the new feature layer created in the last step > Add field > Calculate Field > Divide provider count by population count (Field type is numeric, float, with 15 sig. digits and “show thousands separators”. Calculate by python expression “Provider Count/Total Population”)	Calculates provider to population ratio within each catchment area from each clinic location
Perform join to add the provider to population ratio to the point layer representing all clinic locations	Analysis > Geoprocessing > Join > Load first catchment polygon layer as the “Target Feature” and the outpatient clinic locations as the “Join Features.”	Joins the provider count calculated in the prior step to the data set containing the point feature layer for all outpatient clinics
**Step 2: Accessibility Index of population calculation**		
Run the second catchment area from each census tract centroid	Steps: Analysis Tab > Network Analysis > Service Area > Import Facilities > Set Driving Time to “away from facilities” and “15 minutes,” > Settings to “generalized,” “overlap, > Click	Generates a polygon feature layer
Run spatial join to join second	Analysis > Geoprocessing > Spatial Join > Set Target Feature to the Feature	A new feature layer that has joined the second

catchment area with all clinic locations within 15-minute drive time	Polygon Layer generated from second catchment area, Join Feature to the clinic location feature layer, choose “Join one to one,” and “keep all target features,” set match option to “intersect,” hover over field “provider count” and click “edit,” and then click “sum” > Click Run	catchment area to clinic location data layer
Perform join to add the provider to population ratio to the polygon layer representing census tracts	Analysis > Geoprocessing > Join > Load second catchment polygon layer as the “Target Feature” and the census tract feature layer as the “Join Features.”	A feature layer that includes census tract polygon data as well as accessibility scores.
Feature layer containing all census tract polygon data and accessibility scores were used to create descriptive maps and statistical analysis.		

2.3 Hot Spot Analysis
Hot and cold spots of accessibility were identified using the Getis-Ord Gi* statistic in ArcGIS Pro. A spatial weights matrix was constructed with a 4,800-meter fixed-distance band to approximate urban block distances, and a minimum of two neighbors was assigned to tracts without a neighbor within that distance to accommodate rural areas. Manhattan distance was used to better approximate urban road layouts. False Discovery Rate (FDR) correction was applied to control for multiple testing.

3. Statistical Analysis Details
3.1 R Software Version and Packages
Analyses were conducted in R using the following packages loaded in the script: readxl, stringr, dplyr, and MASS. The script calls session-level functions only implicitly; version numbers were not printed in the output.

3.2 Code Snippets for Recoding Gi_Bin and Model Specification

```
# Import packages and data
library(readxl)  # import excel
library(stringr) # string replace
setwd("/Users/madelineratoza/Desktop")
Hotlog <- read_excel("/Users/madelineratoza/Desktop/Hotspotlogreg.xlsx")

# Subset to 99%, 95%, and 90% hot and cold spots
new_Hotlog <- subset(Hotlog, Hotlog$Gi_Bin == "3" | Hotlog$Gi_Bin == "-3" | Hotlog$Gi_Bin
== "2" | Hotlog$Gi_Bin == "-2" | Hotlog$Gi_Bin == "1" | Hotlog$Gi_Bin == "-1")

# Recode Gi_Bin: 1 = hotspot; 0 = coldspot
library(dplyr)
```

```
new_Hotlog <- new_Hotlog %>% mutate(Gi_Bin = case_when(
  Gi_Bin == 3 ~ 1,
  Gi_Bin == 2 ~ 1,
  Gi_Bin == 1 ~ 1,
  Gi_Bin == -1 ~ 0,
  Gi_Bin == -2 ~ 0,
  Gi_Bin == -3 ~ 0,
  TRUE ~ Gi_Bin
))

# Logistic regression models and stepwise selection
library(MASS)
base.model <- glm(Gi_Bin ~ 1, data = new_Hotlog, family = binomial)
scope.model <- glm(Gi_Bin ~ percent_hispanic + percent_white + percent_black +
  percent_american_indian + percent_asian + percent_native_hawaiian +
percent_other_race +
  percent_two_or_more_races + percent_minority + percent_households_below_povert +
  percent_no_vehicle_households + percent_households_no_partner_p +
  percent_households_single_paren + percent_owns_smartphone +
  percent_no_internet_households + percent_with_disability + percent_unemployed +
  percent_no_hs_diploma, data = new_Hotlog, family = binomial)

step.model <- stepAIC(scope.model, direction = "both", trace = FALSE)
summary(step.model)
```

3.3 **Table S5.** Logistic regression output (coefficients on log-odds scale)

Predictor	Estimate (β)	Std. Error	z value	Pr(>|z|)
(Intercept)	-9.33899	3.98127	-2.346	0.018990
percent_hispanic	0.13595	0.04129	3.293	0.000992
percent_white	0.10746	0.04098	2.622	0.008736
percent_black	0.09932	0.04089	2.429	0.015129
percent_american_indian	0.37082	0.25724	1.442	0.149425
percent_asian	0.11077	0.04178	2.651	0.008014
percent_no_vehicle_households	-0.07500	0.02605	-2.879	0.003988
percent_households_no_partner_p	-0.04543	0.01103	-4.121	3.77e-05
percent_households_single_paren	0.12360	0.02746	4.501	6.77e-06
percent_no_internet_households	0.03715	0.01912	1.943	0.052063
percent_with_disability	0.16974	0.03080	5.511	3.56e-08
percent_no_hs_diploma	-0.07290	0.01943	-3.752	0.000175

Model fit (as printed by summary(step.model)): Null deviance = 522.64 on 438 df; Residual deviance = 371.64 on 427 df; AIC = 395.64; 1 observation deleted due to missingness. Number of Fisher Scoring iterations: 5.

3.4 Residual diagnostics
Model diagnostics were evaluated using null deviance, residual deviance, Akaike Information Criterion (AIC), and comparison across forward, backward, and bidirectional stepwise specifications.

During forward model selection, inclusion of both percent_white and percent_minority resulted in model singularity, with the minority coefficient returned as undefined (NA). This indicated linear dependency among racial composition predictors. Based on this diagnostic finding, percent_minority was excluded from interpretation in the final model, and the final bidirectional and backward stepwise models converged on the same stable predictor set.

3.5 Model selection comparison (AIC)
AIC values printed by AIC(base.model, forward.model, backward.model, step.model):

Table S6. Model selection comparison (AIC)

Model	df	AIC
base.model	1	527.1626
forward.model	18	405.0939
backward.model	12	395.6428
step.model	12	395.6428

Figure S3. This scatterplot illustrates the near-perfect inverse relationship between tract-level percent White and percent minority composition. As expected, increases in percent White correspond directly to decreases in percent minority, producing an essentially linear dependency between the variables. This relationship helps explain the singularity encountered when both variables were considered simultaneously in the regression model.

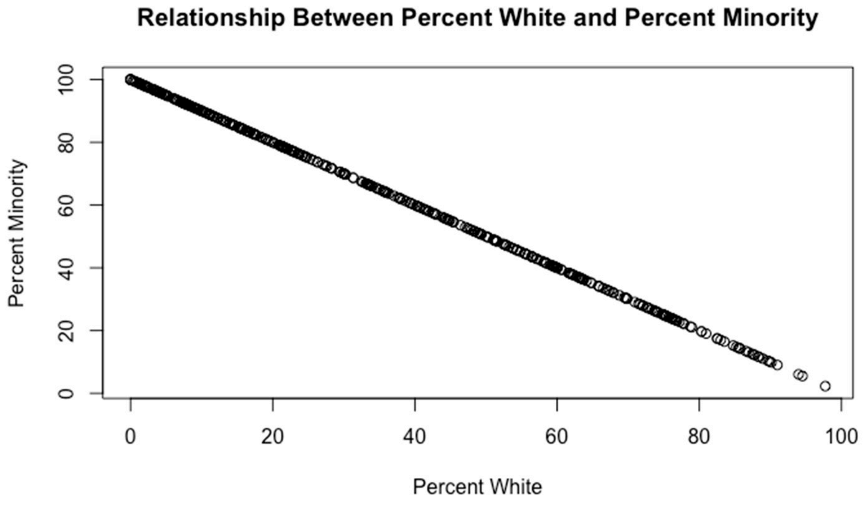

4. Reproducibility Statement

All analyses were performed using ArcGIS Pro (version 3.3, ESRI) with the Network Analyst and Spatial Statistics toolboxes, and R (version 4.3.1) for regression modeling. Publicly available data from the Texas Health and Human Services licensure database (clinic locations) and the 2020 U.S. Census American Community Survey (community characteristics) were used, supplemented with therapist licensure addresses obtained through a state open records request. Road network routing relied on ESRI StreetMap Premium, which is available under commercial license. All data-cleaning workflows, ArcGIS parameters, and R code used for statistical analysis are documented in this Supplement, enabling replication of study findings by researchers with access to the same datasets.
